# Assessing the population structure and genetic variability of Kenyan native goats under extensive production system

**DOI:** 10.1038/s41598-024-67374-2

**Published:** 2024-07-16

**Authors:** Nelly Kichamu, George Wanjala, Ludovic Toma Cziszter, Péter Strausz, Putri Kusuma Astuti, Zoltán Bagi, Szilvia Kusza

**Affiliations:** 1 Centre for Agricultural Genomics and Biotechnology, University of Debrecen, Debrecen, 4032 Hungary; 2 Doctoral School of Animal Science, University of Debrecen, Debrecen, 4032 Hungary; 3 Ministry of Agriculture Livestock, Fisheries and Cooperatives, Directorate of Livestock Development, Naivasha Sheep and Goats Breeding Station, P.O. Box 2238-20117, Naivasha, Kenya; 4 Ministry of Agriculture, Livestock, Fisheries, Cooperatives and Irrigation, Directorate of Livestock Production, P.O. Box 437-50200, Bungoma, Kenya; 5 Faculty of Bioengineering of Animal Resources, University of Life Sciences “King Mihai I” From Timișoara, 300645 Timișoara, Romania; 6 Institute of Strategy and Management, Department of Management, Corvinus University of Budapest, Budapest, 1093 Hungary

**Keywords:** Ecology, Genetics

## Abstract

Indigenous goats are important to many livelihoods. Despite this, they are subjected to indiscriminate crossbreeding. This affects their genetic variability which is needed to survive in current regime of climate change. The study assessed population structure and genetic diversity of Galla and Small East African goats (SEA) using pedigree information. A total of 7384 animals, 5222 of the Galla and 2162 of the SEA breeds, born between the years 1983 and 2022, were utilized. Individuals with known parents were defined as reference population. From the results, the maximum generation traced for Galla and SEA populations was 14.6 and 14.5, respectively. However, only 6 and 5 generations for Galla and SEA were complete. Pedigree completeness increased with the increasing number of generations. The average generation interval (*GI*) for Galla and SEA was 3.84 ± 0.04 and 4.4 ± 0.13 years. The average increase in the rate of inbreeding per generation for Galla and SEA was 0.04 and 0.05, with the effective ancestors and founders (*fa/fe*) ratio being same (1.00) for both breeds. Fifty percent (50%) of the genetic variability in the populations was contributed by 3 and 1 ancestor for Galla SEA, respectively. The effective population size (*Ne*) was 5.19 and 4.77 for Galla and SEA. Therefore, the current breeding programs should be changed to avoid future genetic bottlenecks in this population. These findings offer an opportunity to enhance the current genetic status and management of Kenyan native goats and other regions with similar production systems.

## Introduction

In developing countries, rural farmers in small-scale management systems often produce native breeds that are adapted to their respective areas. The success of these farmers depends on the ability of breeds to produce effectively for them to enhance productivity. Structured selection and conservation programs are among the proposed remedies that can help improve the genetic progress of these native breeds^1,2^. One crucial goal of these programs is to maintain genetic diversity within a population^3^. However, this seems not to work, especially with the extensive production system adopted by most goat farmers in the country. This issue is worsened by inadequate information on the application of sustainable genetic improvement programs as well as constraints in financial resources^4^. Genetic diversity refers to the variety of genotypes or phenotypes available in a given population. This includes all the physical, physiological, and behavioural distinctions observed among individuals and populations^5^. Genetic diversity is essential in matters related to population evolution and adaptation to climate change as well as sustainability within population genetic selection^6,7^. Despite the molecular techniques being employed to assess genetic diversity and relationships within and among herds^7–9^, pedigree analysis remains the most convenient and cost-effective method. This is particularly true for tracking changes in genetic variability, once genomic relationships among founder animals are established^10–12^.

**Figure 1:**
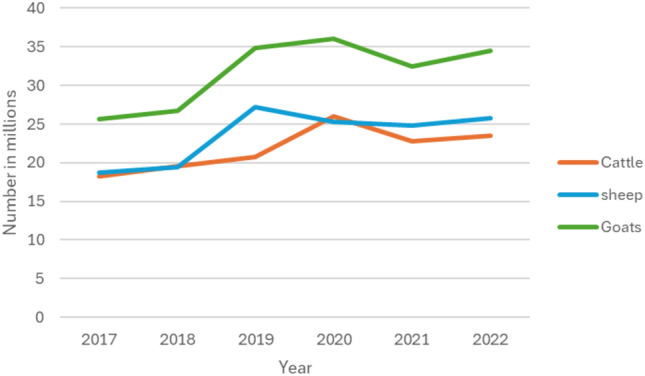
Population trend for goats, sheep and cattle in Kenya. *Source:* FAOSTAT, 2022 ^15^.

Kenya is home to native goat populations widely distributed across diverse agroecological regions^13^. These goats play a vital role in supporting rural household economies by providing food and nutritional security through milk and meat supply, generating income from surplus livestock sales, and serving as insurance against unforeseen risks^1^. Furthermore, they hold significant cultural values and contribute to climate-smart agricultural practices, enhancing farmers' resilience to challenges posed by climate change^1,14^. According to the most recent estimate by^15^, the number of goats was approximately 32.7 million in 2022 which was higher than that of cattle (23.5 million) and sheep (23.8 million) as shown in Fig. 1. This figure emphasizes on how these goats are preferred by most households as compared to sheep and cattle.

**Figure 2:**
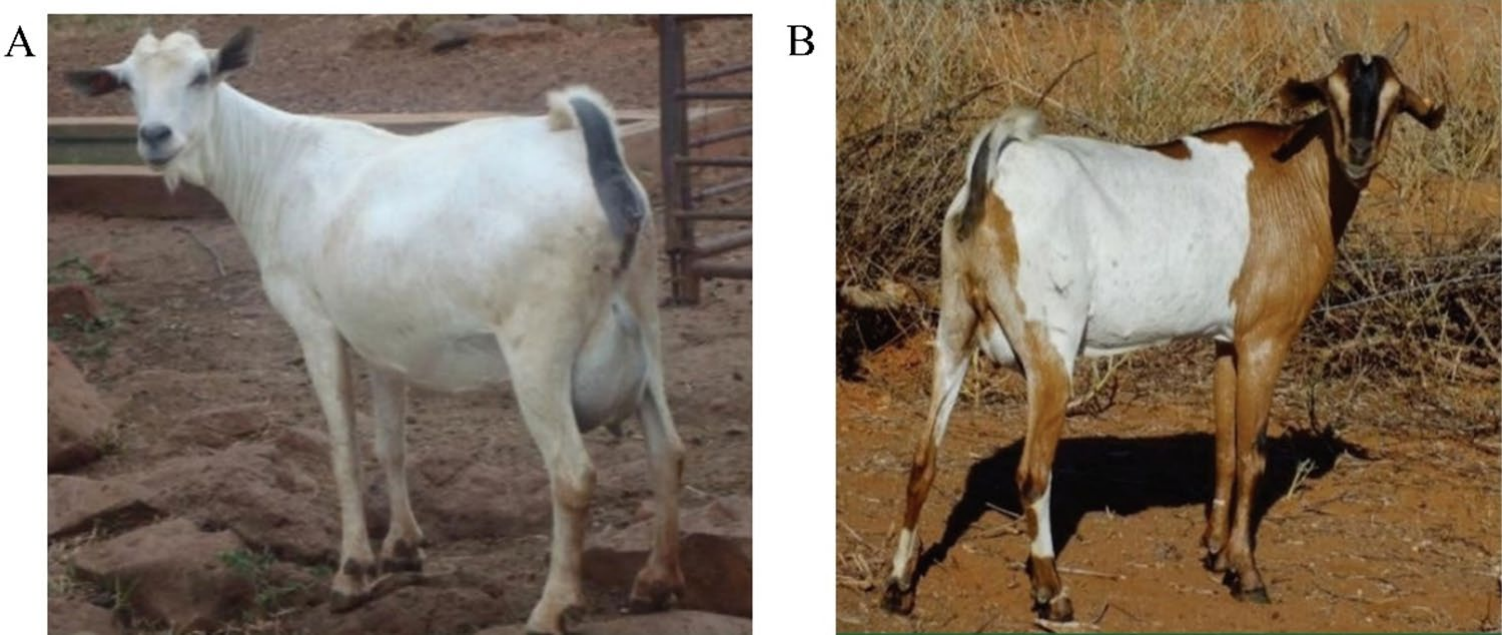
Kenyan Galla (**A**) and Small East African (**B**) native goat breeds.

Among the native goat breeds in Kenya, the Galla goat (Fig. 2A), also known as the Somali or Boran goat, is a hardy breed found in the arid and semi-arid regions of the country. These regions experience minimal precipitation, high temperatures, poor-quality feed supplies, and a high prevalence of livestock diseases^16^. Galla goats are well-adapted to these harsh environmental conditions due to their long and tall body structure. They are predominantly white and primarily raised for meat production, with some communities utilizing them for milk production^17^. The Small East African (SEA) goat breed (Fig. 2B), indigenous to Eastern and Central Africa, is another small ruminant important genetic resource in Kenya. Although highly prolific, hardy, and adaptable to the local environment, SEA goats are less profitable for breeders, as they typically reach a market weight of only 20 kg by the age of 2 years^18^.

Despite their importance, these native breeds have often been neglected by breeders due to their lower productivity and slow growth rate. To improve their performance, the breeds are commonly exposed to indiscriminate crossbreeding with international breeds^13^. However, this practice may affect the adaptive features of these native goats thus leading to extinction or the dilution of their adaptive characteristics^19,20^. To prevent this from happening and ensure the preservation of their valuable traits, it is crucial to establish a sustainable breeding program that will effectively safeguard and manage these traits^14,21^. This can only be achieved by understanding the population structure and genetic variability, using the pedigree or molecular information^7^. Various studies have used molecular data to assess the genetic diversity of these native goats^13,22–25^. But so far, no studies have been done to assess the genetic diversity of Galla and SEA goats using pedigree data which is readily available. This study intends to fill this knowledge gap by examining the population structure and genetic variability of Galla and SEA goats under extensive production system. By evaluating their genetic structure and diversity using the available pedigree data will help in determining ways to develop an effective breeding strategy that aims at conservation and sustainable management of Galla and SEA goats in Kenya.

## Results

### Average number of offspring produced per buck and does

The total number of bucks and does with offspring were 90 and 3063 for Galla and 50 and 1023 for SEA, respectively (Table 1). Among the does that produced offspring only gave rise to an average of 1.61 ± 0.23 kids for Galla and 1.75 ± 0.35 for SEA goats, while the average offspring produced per buck were 59.95 ± 23.2 and 31.16 ± 15.1 for Galla and SEA, respectively. Even though fewer bucks were used than does, there were more replacement offspring born to bucks than to does.

**Table 1:** Number of bucks, does and offspring born by does and bucks, from the period between 1983 and 2022 for Galla and SEA goats.

	Galla				SEA			
	NoB	BoF	NoD	DoF	NoB	BoF	NoD	DoF
Mean	2.73 ± 1.1	59.95 ± 23	92.82 ± 24	1.61 ± 0.2	1.43 ± 0.8	31.16 ± 15	29.23 ± 11	1.75 ± 0.4
Total	90	1978	3063	53	50	1059	1023	61

*NoB* Number of bucks, *BoF* Average number of offsprings born by bucks per period, *NoD* Number of does, *DoF* Average number of offsprings born by does per period.

### Pedigree completeness

**Figure 3:**
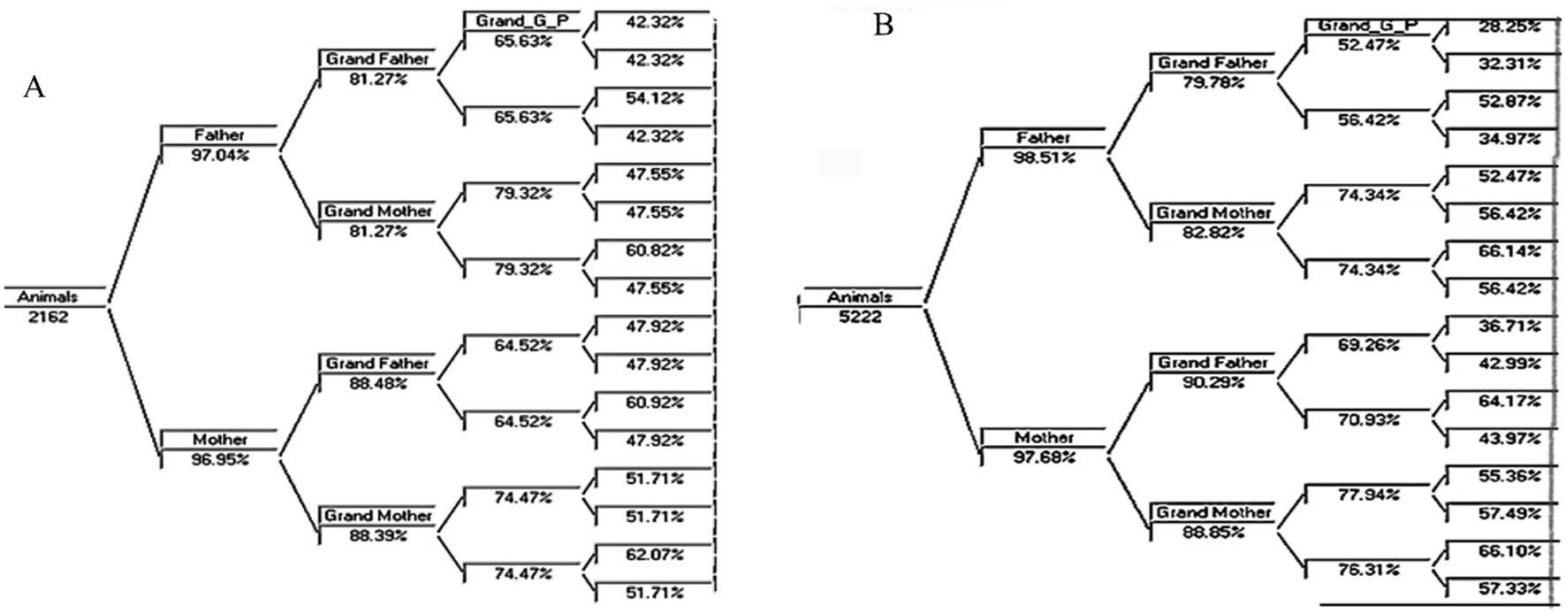
Pedigree completeness for SEA (**A**) and Galla (**B**) goat breeds.

Pedigree completeness has a significant impact on the calculation of the inbreeding coefficient (F). For this study, there were 5,222 and 2,162 animals in the relationship matrix for the Galla and SEA breed populations, respectively. Figure 3 displays the pedigree completeness for both populations. The maximum generation for the Galla and SEA breeds in terms of completeness, was 14.6 and 14.5 respectively. Bucks and does make up the first parental generation, grand-bucks and grand-does make up the second, and great grand doe and great-grand bucks make up the third generation, and so on. For the Galla and SEA breeds, the percentages of first, second, third, and fourth-generation pedigree completeness were 98.09%, 85.44%, 69.00%, 47.34%, and 96.99%, 84.86%, 62.9%, 44.3%, respectively. Figure 3A,B illustrate how the pedigree completeness increased as the maximum generation increased.

### Effective population size (Ne)

The estimates for the mean MG, CG, and EqG for Galla and SEA were 6.9, 2.64, 4.01 and 6.41, 2.91, 4.04, respectively. Their corresponding inbreeding rates (*ΔF*) were 0.04, 0.13, 0.1% for Galla and 0.05, 0.15, 0.10 for SEA breed, respectively (Table 2). The upper and lower limits of Ne for the pedigreed population for Galla and SEA were 3.74, 5.19 and 3.35, 4.77, respectively.

**Table 2:** Inbreeding rate (*ΔF*) and effective size (*Ne*) by type of generation for the Galla and SEA breeds.

Type of generation	Galla		SEA	
	ΔF	Ne	ΔF	Ne
Complete	0.13	3.74	0.15	3.35
Maximum	0.04	8.56	0.05	8.73
Equivalent	0.10	5.19	0.10	4.77

#### Inbreeding coefficient (*F*)

For Galla and SEA, the average inbreeding coefficient was 0.75 and 1.07, respectively. SEA recorded a higher F as compared to Galla goats. For both breeds, F increased from second maximum generation (0.19%) to fourteen maximum generation (0.79%) in Galla goats and from second maximum generation (0.21%) to fourteen maximum generation (0.83%) in SEA. For Galla goats, the proportion of inbred animals in each generation increased as the number of generations grew (Table 3), while the proportion of inbred animals in SEA reached 100% from the 6^th^ generation and remained constant until the 14^th^ generation (Table 4). The mean AR for both breeds increased with generation (*CG*), with Galla recording 0.04 in generation zero (animals with no parental records) and 0.25 in generation six (Table 3), and SEA recording 0.02 in generation zero and 0.82 in generation five (Table 4).

**Table 3:** Population parameters for maximum and complete generations for Galla goats.

G	Maximum generation (*MG*)							Complete generation (*CG*)					
	No	F	∆F	% Inbred	F for inbred	AR	Ne	No	F	% Inbred	F for inbred	AR	Ne
0	77	0				0.01		122	0			0.04	
1	336	0	0.09			0.05		1072	0.17	64.83	0.267	0.10	2.8
2	427	0.19	0.03	21.56	0.22	0.07	2.5	1377	0.41	100	0.41	0.20	1.7
3	398	0.23	0.13	23.62	0.24	0.10	14.5	1309	0.56	100	0.56	0.24	1.9
4	230	0.35	-0.01	34.97	0.35	0.12	3.1	806	0.65	100	0.65	0.26	2.4
5	411	0.34	0.08	34.01	0.34	0.12		416	0.71	100	0.71	0.35	2.8
6	411	0.42	0.04	42.61	0.43	0.12	4.4	120	0.68	100	0.68	0.25	
7	493	0.49	0.05	50.33	0.50	0.18	3.8						
8	462	0.59	0.00	59.20	0.59	0.22	2.6						
9	437	0.59	0.03	60.87	0.61	0.27	48.6						
10	474	0.65	0.04	100	0.65	0.27	3.5						
11	287	0.69	0.00	100	0.69	0.27	3.9						
12	270	0.69	0.06	98.52	0.69	0.34							
13	199	0.75	0.04	100	0.75	0.33	2.8						
14	81	0.79		100	0.79	0.35	3.0

**Table 4:** Population parameters for maximum and complete generations for SEA goats.

G	Maximum generation (*MG*)							Complete generation (*CG*)					
	No	F	% Inbred	∆F	F for Inbred	AR	Ne	No	F	% Inbred	F for inbred	AR	Ne
0	64	0	0		0.01	0		66	0			0.02	
1	184	0	0	0	0.09	0	2.3	340	0.14	46.18	0.31	0.26	3.3
2	115	0.20	100	0.20	0.28	0.20		428	0.22	69.86	0.32	0.39	5.5
3	321	0.16	66.67	− 0.04	0.26	0.25	2.4	460	0.42	100	0.42	0.45	1.9
4	199	0.37	100	0.21	0.41	0.37		619	0.58	100	0.58	0.77	1.7
5	182	0.34	87.91	− 0.03	0.45	0.39	2.3	249	0.70	100	0.70	0.82	1.8
6	56	0.50	100	0.16	0.67	0.50	3.9						
7	115	0.56	100	0.06	0.76	0.56							
8	126	0.42	100	− 0.14	0.68	0.42	4.3						
9	174	0.61	99.43	0.19	0.78	0.61							
10	162	0.59	100	− 0.02	0.78	0.59	4.8						
11	214	0.65	100	0.06	0.80	0.65	4.6						
12	173	0.69	100	0.04	0.82	0.99	5.8						
13	75	0.71	100	0.02	0.81	0.71	1.2						
14	2	0.80	100	0.09	0.85	0.83							

Complete generations (CG) are the number of generations, n, between the individual and the furthest generation when both generations ancestors are known.

Maximum generation (MG) is the number of generations separating the progeny from its most distantly known ancestor along each path.

Generation (G), Numbers (No), average inbreeding coefficients (F%), percentage of inbred individuals (% inbred), average F for inbred animals (F for inbred), average coefficients of relatedness (AR), and effective size (Ne).

Increase in inbreeding (IF),

Increase in inbreeding per generation (∆F).

#### Generation interval (*GI*)

The generation intervals were obtained from four pathways including buck-son (*Lbs*), buck-daughter (*Lbd*), doe-son (*Lds*), and doe-daughter (*Ldd*) are shown in Table 5. The mean generation interval (*GI*) from the four pathways obtained was 3.8 and 4.4 years for Galla and SEA and the mean age of parents at the birth of their offspring was 3.94 ± 0.02 years for Galla and 4.56 ± 0.043 years for SEA (Table 5).

**Table 5:** Generation interval and the average age of parents at the birth of offspring for four different paths for Galla goats and SEA.

Generation interval in years					Average age of the parent at the birth of offspring (years)			
Pathway	Galla goat		SEA		Galla goat		SEA	
	No	Interval	No	Interval	No	Mean age ± SD	No	Mean age ± SD
Buck-Son	18	6.44 ± 0.36	11	4.95 ± 0.74	1814	4.22 ± 0.05	837	4.82 ± 0.07
Buck-Daughter	1342	3.94 ± 0.05	503	4.55 ± 0.09	3313	4.19 ± 0.03	1255	4.64 ± 0.06
Doe-Son	19	3.95 ± 0.47	11	4.75 ± 0.97	1808	3.68 ± 0.05	836	4.55 ± 0.10
Doe-Daughter	1312	3.69 ± 0.06	502	4.39 ± 0.12	3276	3.66 ± 0.04	1254	4.26 ± 0.07
Total	2691	3.83 ± 0.04	1027	4.39 ± 0.12	10,211	3.94 ± 0.02	4182	4.56 ± 0.04

### The effective number of founders (ƒe) and ancestors (ƒa)

Table 6 displays the outcomes of the analysis conducted on the probability of the origin of genes. Galla breed had a higher number of founders as compared to SEA breed, 99 versus 61. The effective number of ancestors *(ƒa)* and the effective number of founders *(ƒa)* were equal in both breeds, with Galla having *f*_*e*_ = 7, and *f*_*a*_ = 7, and SEA having *f*_*e*_ = 2, and *f*_*a*_ = 2). This resulted in the same *f*_*a*_*/f*_*e*_ ratio equal to 1, in both breeds. Number of ancestors explaining 50% of genetic diversity were 3 in Galla breed and 1 in SEA breed.

**Table 6:** Gene origin for Galla and SEA breeds.

Parameters	Galla	SEA
Total number of animals	5222	2162
Animals with both parents known	5100	2096
Animals with one unknown parent	99.5	65
Animals with unknown parents	122	66
Number of founders *(ƒ)*	99	61
The effective number of founders *(ƒe)*	7	2
The effective number of ancestors *(ƒa)*	7	2
No. of ancestors explaining to 50%genetic variability	3	1
Ratio *ƒe/ƒ*	0.07	0.03
Ratio *ƒa/ƒe*	1	1

**Figure 4:**
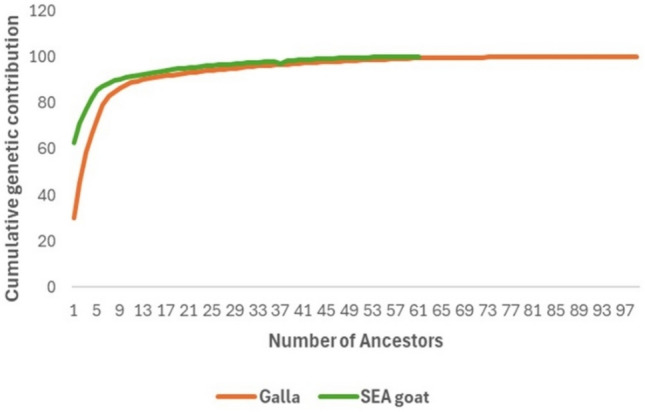
Cumulative marginal contribution of the most important ancestors in studied goat breeds.

Figure 4 shows the cumulative genetic contributions of Galla and SEA goat populations. From the results, both breeds recorded a small number of ancestors which contributed to their genetic pool. In Galla goats, the top three ancestors contributed less than 29.95% of genes. The most important ancestor had 100% contribution. For SEA goats, the least important ancestor made up 62.8% of genes, and the top ancestor had 99.98% impact. This graph also shows that each ancestor had a similar impact in both breeds, except for the first three in Galla goats. Notably, in the Galla goat population, 99 ancestors contributed to 100% of the genetic pool while only 61 ancestors in SEA goats made up 99.98% of genes.

## Discussion

Pedigree completeness is an important parameter to be considered in any population. Previous studies have indicated that pedigree completeness significantly impacts the accuracy of inbreeding coefficient (*F*) calculations and the assessment of inbreeding depression in a population. This is because the likelihood of identifying a common ancestor increases with more complete pedigree information^26^. This is why^27^ emphasizes the importance of having reliable records of the herd in any given study. In this study, we assessed the population structure and genetic diversity of Kenyan native goats using pedigree information.

Pedigree completeness increased as the number of generations increased. This trend may arise because of lack of parental information in the earlier generations (Generation 0). For this study, animals in generation 0 were considered as founder population.

The pattern of pedigree completeness is similar to the estimates reported by^28^ 52%, 10% and 1.4%, for first, second, and third generation were reported by^10^ in Spanish Murciano-Granadina goats. However, for both studies, the level of pedigree completeness increased as the generation increased. The possible reason for this trend was that identification of each animal, pedigree recording, and relationship of each animal were maintained systematically and in an informative way in the nucleus that helped in tracing the pedigree completeness.

The average generation interval (*GI*) was assessed using the four genetic pathways: Buck-son (*Lbs*), Buck-daughter (*Lbd*), Doe-son (*Lds*), and Doe-daughter (*Ldd*). From our study, the SEA goat recorded a higher overall value of generation interval as compared to Galla goats. This could be attributed to the fact that Galla goats tend to have a rapid growth rate as compared to SEA goats which makes them be bred at a younger age. Similar values were reported by^29,30^ for South Khorasan Cashmere and Creole Goats in America, however, a higher generation interval of 5.28 years was reported by^31^ in Brazilian Marota goats. Lower figures for the generation interval of 3.3, 2.77, and 3.3 years were reported by^3,10,28^ in Jamunapari, Spanish Murciano-Granadina and Markhoz goats.

In Galla breed, the generation interval was long for Buck progeny pathways as compared to that of Doe progeny pathway (Lss 6.4, Lsd 3.9, Lds 3.9, Ldd 3.7 years, (Table 5), while SEA recorded higher generation intervals (over 4 years) in all the four pathways. These results are contrary to those reported by^32^ in Raeni Cashmere goats and^28^ in Markhoz goats. The longer interval in Buck-progeny pathway reported in Galla goats can be explained using fewer breeding sires for a long period of time without replacement. Longer intervals for the four pathways in SEA compared to Galla (4.4 vs. 3.8 years) could be the result of the use of Galla Bucks and Does at a younger age due to their fast growth rate. Both scenarios can lead to a reduction in genetic diversity.

Shortening the generation interval is one of the methods that can be applied to increase genetic progress and increase the profitability of the farm. In goat breeding, a GI of up to 3 years can help maximize selection response and genetic variation. Therefore, it is recommended that when developing a breeding program, the does should be allowed to mate for the first time at the average age of 2 years and then, at an interval of 1 year. Bucks should start to be used for breeding at 2 years of age and to be used consecutively for only 2 years^33^.

The effective number of founders (ƒe) and ancestors (ƒa) ratio (*ƒa/ƒe)* varies depending on the genetic structure of the breed, particularly pedigree depth, breeding methods, and the excessive and repeated use of a particular animal^34^. Excessive use of only a few animals for breeding can increase the chances of mating between related individuals if no restrictions are applied. The effective number of founders (*ƒe)* and the contribution of the founders were well-balanced in both breeds, recording similar figures for effective founders(*ƒe)* and ancestors(*ƒa)* with Galla breed having 7 and 7 and SEA breed 2 and 2, respectively. Despite the (*ƒa/ƒe)* ratio being ideal (1.00) for both breeds, 50% of the genetic variability of the population was determined by a few ancestors. Besides, both the breeds had few ancestors who contributed cumulatively to their overall genetic pool. This normally occurs when the selection of breeding animals is based on physical observation where animals with desirable traits are selected for breeding and retained in the farm for a longer period (selective pressure), resulting in a limited genetic diversity in the population.

This scenario may contribute to future genetic bottlenecks if the current mating policy is maintained.

A similar (*ƒa/ƒe)* ratio of was reported by^10^ in Spanish Musiano-Granadina goats. While a higher ratio of 7.10 was reported by^28^ in Iranian Markhoz breed, showing a considerable genetic bottleneck due to the lack of transfer of the alleles to subsequent generations. High or low values of the (*ƒa/ƒe)* ratio always indicate an imbalanced use of Bucks/Does, which poses a risk to the original genetic diversity. When population suffers a demographic decrease,* f*e is overestimated by ignoring some genetic bottleneck effects. Thus^35^, considered that the estimate of the actual number of ancestors (*ƒa)* may have complementary information to (*ƒe)* as this allows us to consider the loss of genetic variation caused by the unbalanced use of sires, which is adequate to evaluate the loss of genetic diversity^10^.

Besides this, effective number of founders (*ƒe)* were lower than the founder numbers (*ƒ)* in both breeds. This may indicate the loss of genetic diversity caused by unbalanced contributions of founders as it was expected that all founders to contribute equally to breeding^36^. The (*ƒe/ƒ)* ratio gives a clear idea about the disequilibrium observed in founders’ contribution to the population. This ratio was found to be 0.07 for Galla and 0.03 for SEA. Different estimates for this ratio were reported by other authors, such as 0.23 in Cashmere goats^37^, 0.46 in Markhoz goats^28^, and 0.14 in Jamunapari goats^3^. These findings further explain the fact that although the main objective of a breeding plan was to select superior genes for breeding purposes, it ultimately led to reducing the genetic diversity of the population, as few animals were used in subsequent generations. These findings also explain the increase in the inbreeding coefficient and the higher average relatedness in the population, along with a decline in genetic diversity and allelic loss due to genetic drift. Hence, this population parameter provides useful information in formulating strategies that will minimize inbreeding in a population.

The coefficient of inbreeding (*F*) measures the number of related alleles that are being produced, the variety of genes that are being lost, and any potential fitness losses linked to a higher risk of phenotypic expression of genetic abnormalities^38^. As a result, it is advised that the level of inbreeding in any population be kept at a manageable level, which is one of the goals of conservation programs^39,40^.

According to^36^, pedigree populations typically have inbreeding rates that are higher than AR rates, because it considers the percentage of a population's whole lineage that descended from a common founder. AR can be used in place of or in addition to inbreeding to predict the long-term inbreeding of a population. As a result, AR can be used to maintain genetic variability in a population by joining animals with low AR values^32^. As AR reaches zero the genetic diversity in a population is said to be maximized.

For this study, the average F and AR for Galla and SEA breeds were 0.46, 0.18 and 0.44, and 0.47, respectively^3^ reported a lower figure of F (0.24) in Jamunapari goats in India. This lower value was due to the periodic introduction of external Bucks that were only allowed to be used for 3 years, and strictly served 20 Does per year. Studies on the genetic variability of Iranian Adani goats also reported a lower F (0.24)^41^. These low values were attributed to the lack of Bucks registration, as approximately 44.4% of animals had missing Buck records. The average increase in inbreeding per generation for Galla and SEA breeds were 0.04 and 0.05. These rates clearly lie within the 1% increase per generation limit set by FAO in assessing breeds classified as threatened with extinction^42^. However, these low inbreeding values can be attributed to missing and inaccurate pedigree information^35^. The average inbreeding coefficient increased over generations indicating that the mating program encouraged breeding amongst closely related animals.

The effective population size (Ne) of between 50 and 100 animals or the annual inbreeding rate of 1% should be maintained for the population to be classified as fit^42^. Further, if it is anticipated that the genetic variability within a population will not increase due to mutation, then an effective population size of 500 animals should be maintained^5^. Reduced effective population size means reduced genetic diversity, and it relates to a variety of negative phenomena, including inbreeding, depression in fitness-related characteristics, and higher fluctuation in selection^8,32^. The Ne reported in the current study for Galla and SEA populations was way below the two limits (50–100) recommended by FAO. Higher findings were reported by^3^ in Indian Jamunapari goats and by^28^ in Markhoz goats. However, missing pedigree information can lead to an overestimation of effective population size suggesting that the population is viable and has an adequate genetic variability, mating can be planned to achieve genetic gain without ignoring the relatedness among animals^43^.

## Conclusions

The Kenyan indigenous goat breeds have been neglected and show inadequate genetic diversity, the study revealed a low effective population size, which is below the FAO's recommended guidelines. This indicates a low genetic diversity among the breeds. Additionally, the research revealed that only a few animals made a noteworthy contribution of 50.00% towards the genetic variation within the breed. Hence, the current breeding program may pose a challenge if not changed. Our findings also show a notable rise in inbreeding and relatedness coefficient which may be underestimated due to the incompleteness of pedigree data and the extensive production system. These factors negatively impact the sustainable breeding of the studied goat populations and can also lead to a reduction in genetic variability. Hence, it is imperative to strike a balance between conservation for sustainable animal breeding and attaining a desirable selection response. Maintaining a proper balance is crucial to uphold genetic diversity and avoid inbreeding, securing the survival and adaptability of our native goat breeds. To ensure the diversity and sustainability of these goat breeds, it is important to create a breeding program which is well coordinated by relevant stakeholders in the field, who can provide useful information to farmers involved in extensive goat breeding, as well as sensitizing them on the importance of conserving the indigenous goats. These findings offer an opportunity to enhance the current genetic status and management of native goats in Kenya and other regions with similar production systems. However, the founder population had missing pedigree information, which could be a limitation to this study. Considering this, future studies should involve the use of molecular data, which can provide a more accurate and comprehensive assessment of the genetic variability of these goat breeds.

## Material and methods

### Animal species and data

**Figure 5:**
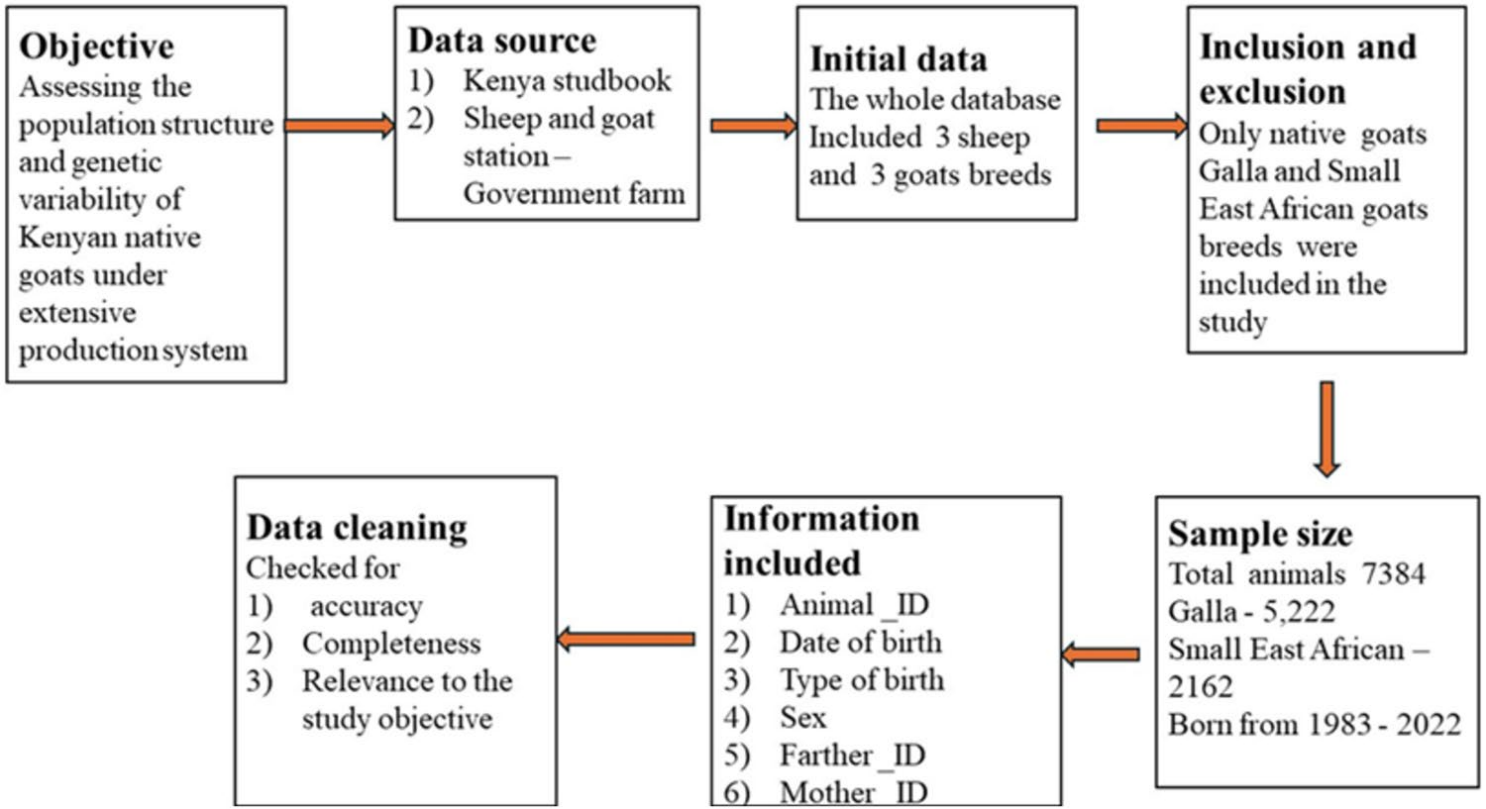
Protocol for data selection and processing flowchart.

Data and pedigree information on Galla and SEA were collected from the Kenya stud book and sheep and goat stations. Figure 5 shows the protocol followed in data selection and processing. A total of 7324 animals (*n* = 5224 Galla and *n* = 2162 SEA) born from 1983 to 2022 were analyzed. Information collected included the animal identification number (Animal_ ID), Doe number (Mother_ ID), Buck number (Father_ ID), Date of birth, and sex of the animal. The flock was maintained under an extensive management system. Animals were given mineral supplements which were always placed next to their sleeping area and were subjected to average daily grazing hours of 7–8, depending on the weather conditions and routine management. The mating period varied across the year with majority falling between April/May and September/October with corresponding kidding occurring in November/December and April/June. The total number of Bucks and Does with offspring were 90 and 3063 for Galla and 50 and 1023 for SEA, respectively. Among the does that produced offspring only gave rise to an average of 1.61 ± 0.23 kids for Galla and 1.75 ± 0.35 for SEA goats, while the average offspring produced per buck were 59.95 ± 23.2 and 31.16 ± 15.1 for Galla and SEA, respectively.

### Statistical analysis

The *ENDOG* software version 4.8^44^ was used to assess various parameters. This software is specifically designed for analyzing populations and genetics using pedigree information. It allows for the assessment of genetic variability and population structure dynamics. The following parameters were analyzed: pedigree completeness, generation interval, inbreeding coefficient, average relatedness, effective population size, effective number of founders and ancestors.

#### Estimating pedigree completeness

The pedigree completeness indicates how much of the lineage is complete in each generation (the number of complete generations known)^45^. This analysis was carried out to determine the accuracy of each individual's pedigree information; (a) Number of maximum traced generations (*MG*), defined as the number of generations separating the progeny from its most distant known ancestor along each path. Those ancestors who did not have parents were regarded as founders and allocated to generation 0, (b) Complete generation equivalent (*EqG*) was calculated as the most distant generation for which all ancestors could be determined, (c) Number of complete generations (*CG*) defined as the number of generations n, between the individual and the furthest generation when both generation ancestors are known and were calculated as:

$$CG = \left( \frac{1}{2} \right)n$$

where n is the number of generations separating the individual to each known ancestor ^30^.

#### Generation interval (GI)

The generation interval was defined as the average age of parents when their offspring are born^38^. GI is important because it helps in the identification of animals to be used for breeding, as those with shorter GI are more likely to pass on their vital traits quickly to the next generation, thereby contributing to quicker genetic progress^28^. In this study, the generation interval was assessed using different pathways: Buck-son *(Lss*), Buck-daughter (*Lsd*), Doe-son (*Lds*), and Doe-daughter (*Ldd*). The average generation interval of the population was calculated by taking the average of the four pathways using the following formula:

$$GI = \frac{Lss + Lsd + Ldd + Lds}{4}$$

#### Inbreeding coefficient (F) and average relatedness coefficient (AR)

The inbreeding coefficient (*F*) is the degree of relationship between two individuals who share common ancestors and was evaluated using the formula proposed by^41^. Inbreeding rates per year (*ΔF/y*) and per generation (*ΔF/g*) were derived by regressing F values on year of birth or EqG, respectively,

The average relatedness coefficient (*AR*) evaluates the probability that any randomly chosen offspring in the population originates from a specific animal in the pedigree data^40^. It could be used in place of or in addition to inbreeding to predict the long-term inbreeding of a population. Therefore, AR can be used to maintain genetic variability in a population by joining animals with low AR values^28^. This coefficient was also computed by employing the methodology described by ^45^.

#### Effective population size (Ne)

Effective population size represents the number of individuals in an ideal population that would result in the same rate of inbreeding as observed in the actual population being studied. It is inversely proportional to the increasing rate of inbreeding^46^. By regressing individual inbreeding on the mean maximum generation (*MG*), mean complete generation (*CG*), and mean equivalent generation (*EqG*), the effective population size (*Ne*) was calculated. This method was preferred as it is used when there is a likelihood of inadequate genealogical data and was done to approximate the lower, higher, and actual bounds of Ne^31^. Individual change in inbreeding ∆Fi was calculated using the formula defined by^40^.

$$\Delta Fi = 1 - \sqrt[{EqGi - 1}]{1 - Fi}$$

where *F*_*i*_ and *E*_*q*_*G*_*i*_ are the coefficient of inbreeding and the equivalent complete generation for the individual *I*, respectively. The coefficients of individual increase in inbreeding were averaged, and realized effective population size was estimated as follows.

$$NeF = {\raise0.7ex\hbox{$1$} \!\mathord{\left/ {\vphantom {1 {2\Delta F}}}\right.\kern-0pt} \!\lower0.7ex\hbox{${2\Delta F}$}}$$

#### The effective number of founders (ƒe) and ancestors (ƒa)

These parameters were analyzed to characterize the genetic variability of the population in terms of gene origin for animals with both parents known (Reference population). The effective number of founders (ƒe) is the number of animals that, if mated randomly, would produce the same amount of genetic variation as the study population. This parameter considered the animals whose genealogy was known. The calculation was performed by summing up the number of progenies of each founder in the population^34^.

$${\text{fe}} = \frac{1}{{\mathop \sum \nolimits_{k = 1 }^{f} \begin{array}{*{20}c} {q2} \\ k \\ \end{array} }}$$

where ƒ was the number of founders and *q*_*k*_ presented the genetic contribution of the* k*^*th*^ founder to the reference population^34^.

The genetic contribution of ancestors (ƒ) of the reference population (founders or not) and their expected marginal contribution to the gene pool were assessed using the method proposed by^34^. This method was estimated using the formula that calculates the likelihood of an offspring inheriting an allele from a specific ancestor proposed by^30^. *ENDOG* software was used for this calculation. while the effective number of ancestors (ƒa), which is the minimum number of ancestors, not necessarily founders, explaining the complete genetic diversity of the current population was also computed basing on marginal contribution of each ancestor as follows:

$${\text{fa}} = \frac{1}{{\mathop \sum \nolimits_{j = 1 }^{a} \begin{array}{*{20}c} {q2} \\ j \\ \end{array} }}$$

where qj is the marginal contribution of an ancestor j, i.e., the genetic contribution made by an ancestor that is not explained by other ancestors chosen previously^30^

### Ethical declarations

There was no ethical approval needed as the data used for the study was from the records kept by the herdbook**.**

## Data Availability

The raw data analysed in this study is the property of the Kenya herd book and sheep and goats project hence are available upon reasonable request through the corresponding author.

## References

[1] Kosgey IS, Rowlands GJ, van Arendonk JAM, Baker RL (2008). Small ruminant production in smallholder and pastoral/extensive farming systems in Kenya. Small Rumin. Res. J. Int. Goat Assoc..

[2] Desta TT (2021). Indigenous village chicken production: A tool for poverty alleviation, the empowerment of women, and rural development. Trop. Anim. Health Prod..

[3] Mandal A, Baneh H, Roy R, Notter DR (2021). Genetic diversity and population structure of Jamunapari goat in India using pedigree analysis. Trop. Animal Health Prod..

[4] Kosgey IS, Baker RL, Udo HMJ, Van Arendonk JAM (2006). Successes and failures of small ruminant breeding programmes in the tropics: A review. Small Rumin. Res.J. Int. Goat Assoc..

[5] Frankham R (2022). Evaluation of proposed genetic goals and targets for the convention on biological diversity. Conserv. Genet..

[6] Boettcher PJ, Hoffmann I, Baumung R, Drucker AG, McManus C, Berg P, Stella A, Nilsen LB, Moran D, Naves M, Thompson MC (2015). Genetic resources and genomics for adaptation of livestock to climate change. Front. Genet..

[7] Sheikhlou M, Abbasi MA (2016). Genetic diversity of Iranian Lori-Bakhtiari sheep assessed by pedigree analysis. Small Rumin. Res. J. Int. Goat Assoc..

[8] Hossam Mahmoud A, Mohammed Abu-Tarbush F, Alshaik M, Aljumaah R, Saleh A (2020). Genetic diversity and population genetic structure of six dromedary camel (camelus dromedarius) populations in Saudi Arabia. Saudi J. Biol. Sci..

[9] Whannou HRV, Spanoghe M, Dayo G-K, Demblon D, Lanterbecq D, Dossa LH (2023). Genetic diversity assessment of the indigenous goat population of Benin using microsatellite markers. Front. Genet..

[10] Oliveira RR, Brasil LHA, Delgado JV, Peguezuelos J, León JM, Guedes DGP, Arandas JKG, Ribeiro MN (2016). Genetic diversity and population structure of the Spanish Murciano-Granadina goat breed according to pedigree data. Small Rumin. Res. J. Int. Goat Assoc..

[11] Galla SJ, Brown L, Couch-Lewis Wheke NWY, Cubrinovska I, Eason D, Gooley RM, Hamilton JA, Heath JA, Hauser SS, Latch EK, Matocq MD, Richardson A, Wold JR, Hogg CJ, Santure AW, Steeves TE (2022). The relevance of pedigrees in the conservation genomics era. Mol. Ecol..

[12] Wu R-S, Wang H-C, Su CL, Wang P-H, Lin E-C (2022). Pedigree-based analyses of changes in genetic variability in three major swine breeds in Taiwan after a disease outbreak. Transl. Anim. Sci..

[13] Waineina RW, Ngeno K, Okeno TO, Ilatsia ED (2021). Genetic diversity and population structure among indigenous and imported goat breeds in Kenya. Genetic Resour..

[14] Mbuku SM, Kosgey IS, Kahi AK (2010). Identification systems and selection criteria for small ruminants among pastoralist communities in northern Kenya: Prospects for a breeding programme. Trop. Anim. Health Prod..

[15] FAOSTAT (2022). http://www.fao.org/faostat/en/#home. Accessed 20 April 2023.

[16] Kahi AK, Wasike CB, Rewe TO (2006). Beef production in the arid and semi-arid lands of Kenya. Outlook Agric..

[17] Mutindi E, Ogali I, Kuria S, Moraa G, Too E, Kingoo J, Ommeh S (2022). Assessment of phenotypes, physiological and behavioural responses associated with heat tolerance among Galla goats in North Eastern Kenya. J. Agric. Sci. Technol..

[18] Safari J, Mushi DE, Mtenga LA, Kifaro GC, Eik LO (2011). Growth, carcass and meat quality characteristics of Small East African goats fed straw based diets. Livestock Sci..

[19] Mwai O, Hanotte O, Kwon Y-J, Cho S (2015). Invited review: African Indigenous Cattle: Unique genetic resources in a rapidly changing world. Asian-Aust. J. Anim. Sci..

[20] Sila W, Gachuiri CK, Recha JW, Audho J, Ojango JMK (2021). Adaptation and returns from improved indigenous small ruminants in climatically challenged smallholder systems of Kenya. Sustainability.

[21] Wanjala G, Kichamu N, Cziszter LT, Astuti PK, Kusza S (2023). An on-station analysis of factors affecting growth traits of pure red maasai and dorper sheep breeds under an extensive production system. Anim. Open Access J. MDPI.

[22] Waineina, R. W., Ngeno, K., Otieno, T. O., & Ilatsia, E. D. Genetic diversity and population structure among goat genotypes in Kenya. *bioRxiv*, 2020–07 10.1101/2020.07.06.189290 (2020).

[23] Tarekegn GM, Wouobeng P, Jaures KS, Mrode R, Edea Z, Liu B, Zhang W, Mwai OA, Dessie T, Tesfaye K, Strandberg E (2019). Genome-wide diversity and demographic dynamics of Cameroon goats and their divergence from east African, north African, and Asian conspecifics. PloS One.

[24] Bertolini F, Servin B, Talenti A, Rochat E, Kim ES, Oget C, Palhière I, Crisà A, Catillo G, Steri R, Amills M, Colli L, Marras G, Milanesi M, Nicolazzi E, Rosen BD, Van Tassell CP, Guldbrandtsen B, Sonstegard TS, Crepaldi P (2018). Signatures of selection and environmental adaptation across the goat genome post-domestication. Genet. Sel. Evol..

[25] Muema, E. K., Wakhungu, J. W., Hanotte, O., & Han, J. Genetic diversity and relationship of indigenous goats of Sub-Saharan Africa using microsatellite DNA markers. https://hdl.handle.net/10568/299 (2009).

[26] Malhado CHM, Malhado ACM, Carneiro PLS, Ramos AA, Ambrosini DP, Pala A (2012). Population structure and genetic variability in the Murrah dairy breed of water buffalo in Brazil accessed via pedigree analysis. Trop. Anim. Health Prod..

[27] Baldursdóttir BK, Kristjansson T, Hallsson J (2012). H (2012) Diversity of the Icelandic goat breed assessed using population data. Acta Agric. Scand. Sect. A Anim. Sci..

[28] Rashidi A, Mokhtari MS, Gutiérrez JP (2015). Pedigree analysis and inbreeding effects on early growth traits and greasy fleece weight in Markhoz goat. Small Rumin. Res. J. Int. Goat Assoc..

[29] Joezy-Shekalgorabi S, Maghsoudi A, Taheri-Yeganeh A, Rajabi-Marand B (2016). Pedigree analysis of Cashmere goat breed of South Khorasan. Italian J. Anim. Sci..

[30] Ginja C, Gama LT, Martínez A, Sevane N, Martin-Burriel I, Lanari MR, Revidatti MA, Aranguren-Méndez JA, Bedotti DO, Ribeiro MN, Sponenberg P, Aguirre EL, Alvarez-Franco LA, Menezes MPC, Chacón E, Galarza A, Gómez-Urviola N, Martínez-López OR, Pimenta-Filho EC, da Rocha LL, Stemmer A, Landi V, Delgado-Bermejo JV (2017). Genetic diversity and patterns of population structure in Creole goats from the Americas. Anim. Genet..

[31] Barros EA, Ribeiro MN, Almeida MJO, Araújo AM (2011). Population structure and genetic variability of the Marota goat breed. Arch. Zootec..

[32] Mokhtari MS, Damaneh MM, Gutierrez JP (2017). Genetic variability and population structure of Raeini Cashmere goats determined by pedigree analysis. J. Livestock Sci. Technol..

[33] Mwangi S, Muasya TK, Ilatsia ED, Kahi AK (2016). Assessment of the genetic variability using pedigree analysis of the Sahiwal breed in Kenya. Ressour. Genet. Anim..

[34] Boichard D, Maignel L, Verrier E (1997). The value of using probabilities of gene origin to measure genetic variability in a population. Genet. Sel. Evol. GSE.

[35] Bernardes PA, Grossi DA, Savegnago RP, Buzanskas ME, Ramos SB, Romanzini EP, Guidolin DGF, Bezerra LAF, Lôbo RB, Munari DP (2016). Population structure of Tabapuã beef cattle using pedigree analysis. Livestock Sci..

[36] Martínez RA, García D, Gallego JL, Onofre G, Pérez J, Cañón J (2008). Genetic variability in Colombian Creole cattle populations estimated by pedigree information. J. Anim. Sci..

[37] Wang Z, Zhou B, Zhang T, Yan X, Yu Y, Li J, Mei B, Wang Z, Zhang Y, Wang R, Lv Q, Liu Z, Zhao Y, Du C, Su R (2021). Assessing genetic diversity and estimating the inbreeding effect on economic traits of Inner Mongolia White Cashmere goats through pedigree analysis. Front. Vet. Sci..

[38] Caballero A, Fernández A, Villanueva B, Toro MA (2022). A comparison of marker-based estimators of inbreeding and inbreeding depression. Genet. Sel. Evol. GSE.

[39] Carolino N, Gama LT (2008). Inbreeding depression on beef cattle traits: Estimates, nearity of effects and heterogeneity among sire-families. Genet. Select. Evol. GSE.

[40] Goyache F, Gutierrez JP, Fernandez I, Gomez E, Alvarez I, Diez J, Royo LJ (2003). Using pedigree information to monitor genetic variability of endangered populations: The Xalda sheep breed of Asturias as an example. J. Anim. Breed. Genet..

[41] Joezy-Shekalgorabi S, Maghsoudi A, Taheri-Yeganeh A, Rajabi-Marand B (2017). Genetic variability of Iranian Adani goat breed using pedigree analysis. J. Animal Plant Sci..

[42] FAO, Secondary Guidelines for Development of National Farm Animal Genetic Resources Management Plans: Page 63 in Management of Small Populations at Risk. https://www.fao.org/documents/card (1998).

[43] Hill WG, Mackay TFC (1996). Introduction to Quantitative Genetics.

[44] Gutiérrez JP, Goyache F (2005). A note on ENDOG: A computer program for analysing pedigree information. J. Anim. Breed. Genet..

[45] Meuwissen T, Luo Z (1992). Computing inbreeding coefficients in large populations. Genet. Sel. Evol..

[46] Gutiérrez JP, Cervantes I, Goyache F (2009). Improving the estimation of realized effective population sizes in farm animals. J. Anim. Breed. Genet..

